# The Unique Roles of Microbial Abundant and Rare Taxa in Regulating Pathogen Dynamics in Wastewater Bioaerosols

**DOI:** 10.3390/microorganisms14010100

**Published:** 2026-01-02

**Authors:** Zhiruo Zhang, Ying Zhang, Qiyu Zhu, Baiheng Qian, Fanyu Ge, Yang Huo

**Affiliations:** 1 Key Laboratory of Songliao Aquatic Environment, Ministry of Education, Jilin Jianzhu University, Changchun 130118, China; zhangzhiruo@jlju.edu.cn; 2 School of Economics and Management, Jilin Jianzhu University, Changchun 130118, China; 3 School of Environment, Northeast Normal University, Changchun 130117, China; zhangy938@nenu.edu.cn (Y.Z.); zhuqy@nenu.edu.cn (Q.Z.); qianbaiheng@nenu.edu.cn (B.Q.); gefy123@nenu.edu.cn (F.G.); 4 Science and Technology Innovation Center for Municipal Wastewater Treatment and Water Quality Protection, Northeast Normal University, Changchun 130117, China; 5 Center for Advanced Optoelectronic Functional Materials Research, Key Laboratory of UV Light-Emitting Materials and Technology of Ministry of Education, Northeast Normal University, Changchun 130024, China

**Keywords:** bioaerosols, abundant taxa, rare taxa, community assembly, network analysis, pathogens

## Abstract

Bioaerosols emitted from wastewater treatment plants (WWTPs) are key vectors for airborne microbial transmission, yet the mechanisms by which abundant and rare microbial taxa regulate pathogen dynamics remain unclear. This study explored the ecological roles of abundant and rare taxa through a comprehensive analysis of bioaerosols from two full-scale WWTPs, integrating high-throughput sequencing of bacterial and fungal communities. Results showed that the rare taxa exhibited higher alpha diversity, and their community construction was dominated by deterministic processes. While the abundant taxa showed higher spatial homogeneity, and their distribution was more consistent with the neutral model, suggesting the dominance of stochastic processes. Network analysis revealed that rare taxa held keystone topological roles within the microbial networks. Moreover, partial least squares path model quantified their direct effects on pathogen abundance, revealing a strong positive direct effect of abundant bacterial taxa but a significant negative direct effect of rare bacterial taxa. This study elucidates the dual roles of taxa with different abundance levels in community assembly and pathogen regulation, emphasizing that effective risk assessment and management strategies should account not only for the carrier role of abundant taxa but also for the regulatory function of the rare biosphere in shaping pathogen dynamics.

## 1. Introduction

Wastewater treatment plants (WWTPs) are critical infrastructures for protecting public and environmental health by removing pollutants and pathogens from municipal sewage [1]. However, the aeration, agitation, and mechanical handling inherent to treatment processes are known to generate significant quantities of bioaerosols, which pose a potential risk for microbial exposure to workers and nearby communities [2,3]. These bioaerosols are complex mixtures of airborne microorganisms, including both commensal environmental bacteria and fungi, as well as human opportunistic pathogens [4,5]. Numerous studies have detected and quantified pathogenic bacteria (e.g., *Pseudomonas*, *Acinetobacter*, *Mycobacterium*) and fungi (e.g., *Aspergillus*, *Penicillium*) in WWTP bioaerosols, linking exposure to respiratory, gastrointestinal, and skin infections among plant operators [6,7]. Consequently, understanding the composition, dynamics, and pathogen load of WWTP bioaerosols is crucial. It is a subject of intense research interest and significant public health concern [8,9].

The microbial community in bioaerosols originates from the complex ecosystems of wastewater and activated sludge, shaped by various factors such as treatment stages, environmental conditions, and microbial interactions [5]. This results in a dynamic assemblage, with some taxa being more abundant and dominant in specific treatment stages, while others remain relatively rare. Conventional microbial ecology has focused on the most abundant taxa, which are thought to dominate community biomass and drive core ecosystem functions like organic matter degradation and nutrient removal [10,11]. In contrast, the rare biosphere is now recognized as a reservoir of immense microbial diversity and genetic potential [12]. Advances in high-throughput sequencing allowed us to directly study the “rare biosphere”. Recent studies reveal that rare taxa can exhibit unique metabolic activities and present distinct biogeography, especially in the regulation of pathogen populations or the maintenance of biodiversity under fluctuating environmental conditions [13]. Although the ecological partitioning between abundant and rare taxa is increasingly recognized in many environmental media [14,15,16], their specific contributions to bioaerosol composition and risk remain poorly understood.

To elucidate how bioaerosol communities form and disperse, it is key to analyze their assembly processes. Microbial communities’ assembly is mainly governed by the balance between deterministic (niche-based) processes (such as environmental screening and interspecific interactions) and stochastic (neutral) processes (including random birth, death, dispersal, and ecological drift) [17]. Recent evidence suggests that the construction of abundant and rare subcommunities may be dominated by different processes. Stochastic processes dominate the abundant groups, while deterministic processes shape the rare biosphere [18]. In addition to community composition, the interaction between microorganisms in bioaerosols also affects the stability and function of the community. Co-occurrence network analysis has become a powerful tool for inferring potential ecological interactions and identifying key groups that have a disproportionate impact on community structure [19]. In engineered ecosystems, rare taxa can occupy a central position in the co-occurrence network, indicating that they have a potential role in maintaining community stability and function beyond their quantitative scarcity [20]. Whether this paradigm is applicable to WWTPs’ bioaerosols and how different treatment units screen these subcommunities are fundamental questions that have not yet been systematically addressed.

The abundance of pathogens in bioaerosols does not only reflect their presence in source sewage, but is more likely to be shaped by the broader ecological context of the entire microbial community [2,21]. Although some studies have linked specific environmental parameters to pathogen levels [5], the clear and potentially contrasting role of abundant and rare subcommunities in promoting or inhibiting pathogen abundance has been largely ignored. The abundant groups may directly promote the aerosolization and concentration increase of pathogens due to their quantitative advantages and potential co-aggregation ability [22]. In contrast, the rare biosphere, as a “microbial seed bank”, may contain key species that can indirectly regulate pathogens through specific antagonistic interactions or by maintaining the stability of the overall community [23]. Clarifying these direct and indirect effects is critical for developing targeted strategies to reduce pathogen emissions.

Based on these knowledge gaps, this study aims to systematically decipher the distinct ecological roles of microbial abundant and rare taxa in shaping bioaerosol communities and regulating pathogen dynamics within full-scale WWTPs. We hypothesized that the distinct assembly processes (stochastic vs. deterministic) governing abundant and rare taxa would lead to their differential roles in the co-occurrence network, and ultimately result in contrasting direct effects of these two subcommunities on airborne pathogen abundance. By testing these hypotheses, this work seeks to provide novel ecological insights into the construction and interactions of the bioaerosol microbiome. The findings are expected to advance the theoretical framework for understanding aerosol microbial community assembly and offer a fresh perspective for assessing and managing microbial exposure risks associated with wastewater treatment, ultimately contributing to a paradigm shift from pathogen-centric monitoring to community-level risk management.

## 2. Materials and Methods

### 2.1. Study Sites and the Sampling Campaign

**Figure 1:**
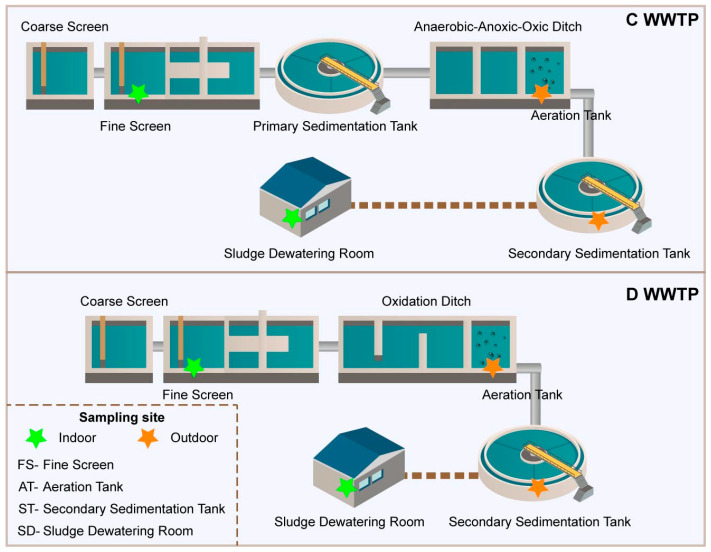
Distribution of the bioaerosol sample sampling sites. Fine screen chamber (FS), aeration tank (AT), secondary sedimentation tank (ST), and sludge dewatering room (SD).

The bioaerosol sampling was conducted in summer (July–August) of 2021 in two municipal WWTPs (Chuanhu and Dongnan, C-WWTP and D-WWTP) located in Changchun, northeast China. The C-WWTP receives influent consisting of approximately 53% municipal sewage and 47% industrial and other wastewater, with a daily treatment capacity of 2 × 10^4^ m^3^. The D-WWTP treats wastewater that is about 71% municipal in origin, with the remaining 29% derived from industrial and other sources, and has a daily capacity of 1 × 10^4^ m^3^. The main treatment processes of C-WWTP and D-WWTP are A^2^O and oxidation ditch, respectively, and both plants employ fine-bubble aeration in their biological treatment units. For each WWTP, we collected bioaerosol samples from key treatment units (fine screen chamber, FS; aeration tank, AT; secondary sedimentation tank, ST; and sludge dewatering room, SD) (Figure 1), and each was set at a height of 1.5 m above the ground, which mimicked the average respiration height of an adult human. The bioaerosols were collected using an Andersen eight-stage air impactor (Andersen, Cleves, OH, USA) and enriched on quartz membranes (90 mm in diameter, Munktell, Falun, Sweden). The air impactor was sterilized by 75% (*v*/*v*) ethanol before and after each sampling, while the membranes were pre-heated for 5 h at 500 °C and placed in sterile storage boxes before use. The bioaerosol samples were continuously collected for 8 h (from 8 a.m. to 4 p.m.) at a flow rate of 28.3 L/min, and the filter membrane was changed every 4 h. A total of 64 aerosol samples were collected, and all samples were stored at −20 °C for subsequent DNA extraction.

### 2.2. Samples Pretreatment and DNA Extraction

The bioaerosol samples were pretreated for improved DNA extraction efficiency, followed by methods described previously [24]. In brief, each filter membrane was cut into fragments and washed with sterilized 1×phosphate-buffered saline (PBS) under low-speed centrifugation (200 *g* at 4 °C for 3 h). The elute was subsequently filtered through 0.22-μm polyethersulfone (PES) membrane disc filters (47 mm in diameter). Bioaerosol DNA extraction was conducted using DNeasy PowerSoil Pro Kit (QIAGEN, Dusseldorf, Germany). The steps of extractions were followed by the standard PowerSoil DNA isolation protocol, except that the column purification process was replaced by bead purification for improved DNA yield (Magic DNA Select beads, Magic-Bio, Hangzhou, China) [24,25].

### 2.3. High-Throughput Sequencing and Bioinformatics Analysis

A total of 54 and 48 qualified bioaerosol samples were, respectively, subjected to analysis of bacterial and fungal community diversity by Illumina high-throughput sequencing (Shanghai MajorBio Bio-pharm Co., Ltd., Shanghai, China). The universal primers 338F/806R for bacteria [2,26] or ITS1F/ITS2 for fungi [27] was used for the amplification and sequencing of the PCR products. The sequencing was performed on an Illumina MiSeq PE300 platform (Illumina Inc., San Diego, CA, USA). Details for amplification and sequencing pipeline can be found in our previous study [28]. Raw sequence data have been uploaded to the NCBI SRA database with the accession numbers PRJNA894540 and PRJNA1392901 for bacteria and fungi, respectively.

The primer cut and pair-end merged reads of each sample were analyzed using EasyAmplicon (https://github.com/YongxinLiu/EasyAmplicon, accessed on 15 January 2025). The high-quality reads were denoised to amplicon sequence variants (ASVs) with the USEARCH method at a similarity threshold of 97%. The taxonomic annotation of bacterial and fungal reads was performed by SILVA v138 [29,30,31] and UNITE v10.05.2021 [32], respectively. To account for variations in sequencing depth, we performed rarefaction on the bacterial and fungal communities by subsampling sequences to 16,058 and 3000 per sample, respectively. This process yielded a final ASV table containing 3918 bacterial ASVs and 1523 fungal ASVs. Microbial genera related with pathogen was identified referred to a previous study [4].

### 2.4. Identification of Abundant and Rare Taxa

**Figure 2:**
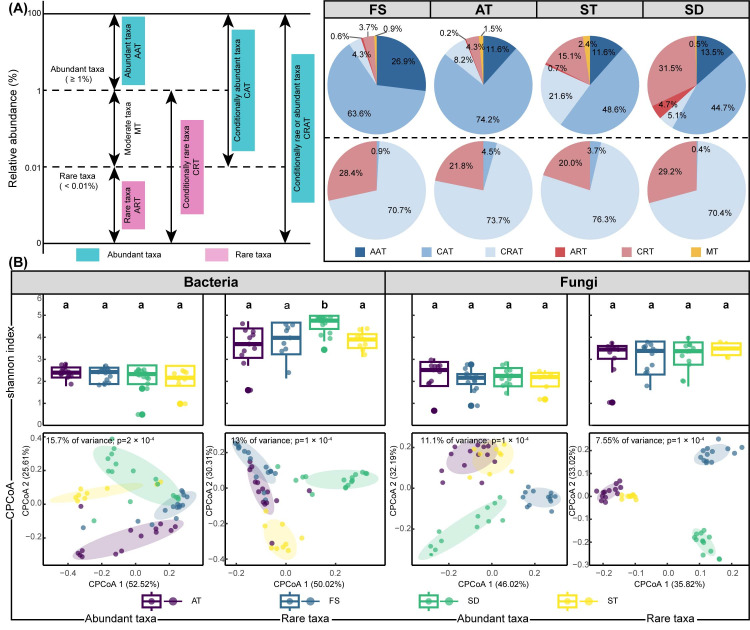
Distribution and diversity of abundant and rare taxa of bioaerosol samples. (**A**) Distribution characteristics of abundant and rare taxa, (**B**) Shannon index and CPCoA of abundant and rare taxa. The letters above the box plot represent the significant level of difference between groups. AAT: abundant taxa, CAT: conditionally abundant taxa, CART: conditionally rare and abundant taxa, ART: always rare taxa, CRT: conditionally rare taxa, MT: moderate taxa.

To investigate the differences in ecological strategies of subcommunities in bioaerosol samples, ASVs were separated into six categories in terms of their relative abundances (Figure 2A) [33]. Always abundant taxa (AAT): abundance ≥ 1% in all samples, always rare taxa (ART): abundance < 0.01% in all samples, moderate taxa (MT), abundance between 0.01 and 1% in all samples, conditionally rare taxa (CRT): abundance below 1% in all samples and <0.01% in some samples, conditionally abundant taxa (CAT): abundance ≥ 0.01% in all samples and ≥1% in some samples but never rare (<0.01%), and conditionally rare and abundant taxa (CRAT): abundance varying from rare (<0.01%) to abundant (≥1%). In this study, we artificially combined abundant taxa (AAT), conditionally abundant taxa (CAT), and conditionally rare and abundant taxa (CRAT) as abundant taxa, always rare taxa (ART) and conditionally rare taxa (CRT) as rare taxa [14,34].

### 2.5. Statistical Analysis

Statistical analysis was performed in R version 4.1.2. Community alpha diversity was estimated using the Shannon index. Differences in community composition (beta diversity) were tested using canonical principal coordinate analysis (CPCoA). To infer the relative role of stochastic processes on community assembly, we fitted the Sloan neutral community model (NCM) [17,35]. Potential ecological associations were inferred by constructing co-occurrence networks based on Spearman correlation (|r| > 0.8, *p* < 0.05), and co-occurrence patterns were visualized using ggClusterNet (v0.1.0) [36] and Gephi 0.9.2. Finally, to quantify the direct and indirect effects among microbial subcommunities and pathogen abundance, we employed partial least squares path modeling (PLS-PM).

## 3. Results and Discussion

### 3.1. Abundant and Rare Taxa in the Aerosol Microbial Communities

To decipher microbial dynamics and their ecosystem roles, we categorized taxa in WWTP bioaerosols as abundant or rare. The pie charts for each site-fine screen chamber, aeration tank, secondary sedimentation tank, and sludge dewatering room-reveal varying proportions of abundant taxa, indicating differences in community dominance among sites (Figure 2A). The higher proportion of abundant taxa and conditionally abundant taxa in aeration tank for bacteria indicates a potentially more stable and predictable microbial community, which might be beneficial for maintaining efficient wastewater treatment processes. Aeration tank provides high oxygen and organic load, favoring the growth of certain dominant bacterial phyla such as *Proteobacteria* and *Bacteroidetes*. The distribution of conditionally abundant taxa across sites shows a more varied pattern, with sludge dewatering room having a notably higher proportion (44.7%), indicating a greater presence of conditionally abundant taxa in this environment.

Alpha diversity, assessed using the Shannon index, was significantly higher for rare taxa compared to abundant taxa across all treatment units (Figure 2B). This pattern aligns with observations in other environmental microbiomes [37]. This suggests that rare taxa contribute disproportionately to the taxonomic richness and evenness within the bioaerosol microbiome. The higher diversity of rare taxa may serve as a reservoir of genetic and functional potential, enhancing the community’s ability to respond to environmental fluctuations [12]. In contrast, abundant taxa, though fewer in number of ASVs, accounted for a substantial proportion of the total sequence reads, indicating their significant contribution to community biomass. This dichotomy between richness and abundance highlights the distinct ecological strategies employed by different microbial groups within bioaerosol communities. CPCoA revealed distinct clustering patterns between abundant and rare taxa (Figure 2B). The community composition of both abundant and rare taxa varied significantly across different treatment units, suggesting that local environmental conditions and operational parameters at each unit play a crucial role in shaping microbial assembly. Notably, the dispersion of abundant taxa communities was greater than that of rare taxa, indicating higher spatial heterogeneity and niche differentiation among abundant members [38]. Aerosol samples from sludge dewatering room had the highest diversity and were significantly different from the microbial community composition of other treatment units.

Recent studies have emphasized the importance of considering both abundant and rare taxa for a comprehensive understanding of ecosystem functioning and stability [39]. In WWTP bioaerosols, the higher diversity of rare taxa could potentially enhance functional redundancy and resilience, buffering the community against perturbations such as changes in temperature, humidity, or chemical exposure [40]. Furthermore, some rare taxa might possess unique functional traits, including the ability to degrade recalcitrant pollutants or inhibit pathogens, which are not prevalent in the abundant core [41]. Their activation under specific conditions could significantly alter community dynamics and function. The observed patterns can be attributed to the different ecological niches and life-history strategies of abundant and rare taxa. Abundant taxa, often considered habitat generalists or opportunists, likely thrive due to their competitive superiority under the relatively stable and nutrient-rich conditions of specific WWTP units. The vast diversity of rare taxa may represent a seed bank of functionally diverse organisms, including specialists and transient members originating from the wastewater itself or the surrounding air [42]. Understanding the dynamics of these subcommunities is crucial for assessing microbial ecology in WWTPs and its implications for bioaerosol dispersion, ecosystem health, and potential human pathogen exposure.

### 3.2. Community Assembly Mechanism of Abundant and Rare Taxa

**Figure 3:**
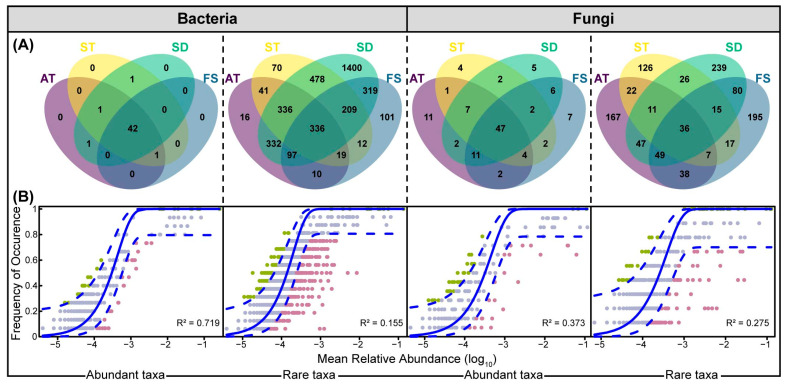
Venn diagram of shared and unique ASVs (**A**); Fit of the Sloan neutral community model (NCM) to ASVs (**B**). The solid blue lines indicate the best fit to the NCM and the dashed blue lines represent 95% confidence intervals around the model predictions. ASVs that occur more and less frequently than predicted by the NCM are shown in different colors. R^2^ indicates the fit of the model.

Venn analysis revealed stark contrasts. Abundant taxa showed high species overlap across units, with few unique ASVs, suggesting a core group of generalists. In contrast, each unit harbored a substantial proportion of unique rare ASVs, indicating a highly differentiated rare biosphere (Figure 3A). Specifically, the abundant taxa exhibited an exceptionally high degree of species overlap across the four treatment units, with each unit harboring very few unique ASVs. This was particularly evident in bacterial communities, where the core set of abundant ASVs was nearly identical regardless of the sampling location. While each treatment unit possessed a substantial proportion of unique rare ASVs, indicating that the rare biosphere was highly differentiated across the plant’s compartments. This clear taxonomic segregation suggests that the abundant and rare taxa occupy distinct ecological niches within the WWTP bioaerosol environment, potentially driven by different adaptive strategies and resource utilization patterns [10]. The homogeneity of abundant taxa suggests they represent a core group of habitat generalists. These taxa likely possess broad metabolic capabilities that allow them to thrive across the varying conditions of different treatment units. Their pervasive presence implies they are well-adapted to the common selective pressures of the WWTP environment and may be efficiently dispersed via aerosolization throughout the facility. Conversely, the high heterogeneity of the rare biosphere indicates a strong role for habitat specialists [37]. The unique environmental conditions at each unit act as distinct environmental filters [19], which deterministically select for specific sets of rare taxa adapted to these micro-niches, leading to the observed high beta diversity.

To quantitatively assess the forces driving these assembly patterns, we fitted NCM to both abundant and rare taxa. The model fit (R^2^) was significantly higher for abundant taxa compared to rare taxa in both bacterial and fungal communities (Figure 3B). A higher R^2^ indicates that neutral processes, such as random dispersal, extinction, and ecological drift, play a dominant role in governing the distribution and occurrence of abundant taxa [43]. This aligns perfectly with their observed homogeneity; their presence across units can be largely predicted by chance and dispersal, rather than strong environmental selection [22]. Conversely, the poorer fit of the NCM to the rare taxa underscores the primacy of deterministic processes in their assembly. Environmental parameters exert a powerful selective pressure, filtering and shaping the rare community in a unit-specific manner. This deterministic control explains why the rare taxa composition differs significantly from one unit to another. Our findings are consistent with emerging paradigms in microbial ecology, which posit that while abundant taxa are often widely dispersed, rare taxa are more constrained by environmental factors, leading to higher spatial turnover and greater sensitivity to environmental change [40].

### 3.3. Co-Occurrence Network Analysis of Microbial Abundant and Rare Taxa

**Figure 4:**
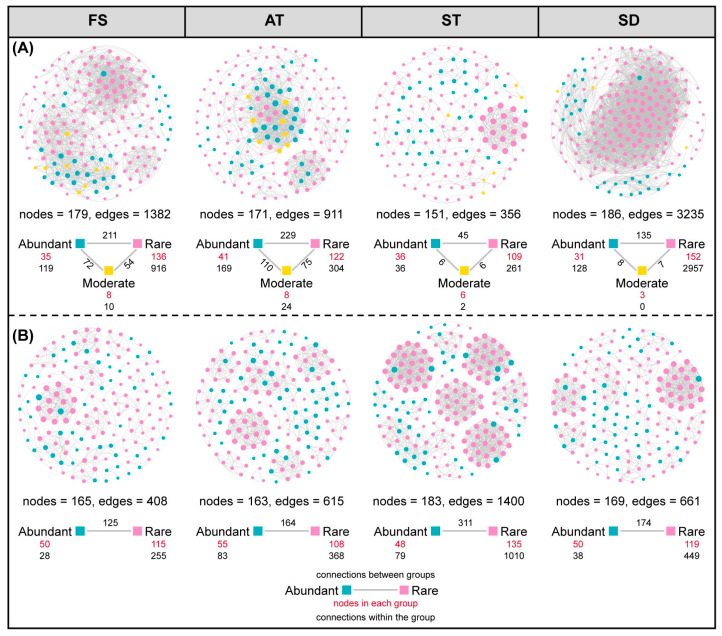
Co-occurrence network analysis revealed the coexistence patterns between abundant and rare taxa in the aerosol bacterial (**A**) and fungal (**B**) communities. Spearman’s correlation coefficient (|r| ≥ 0.8 and *p* < 0.05). The size of each node is proportional to the node degree of the ASVs. The black numbers below each taxa group indicate the number of connections within the group, and the black numbers between colored blocks indicate connections between groups. Red italicized numbers indicate the number of nodes in each group.

To elucidate the intricate ecological interactions within the bioaerosol microbiome, networks were constructed based on Spearman’s correlation coefficient (|r| > 0.8 and *p* < 0.05), indicating strong positive correlations between abundant and rare taxa (Figure 4). In the bacterial community (Figure 4A), fine screen chamber shows a high number of abundant taxa (211) with a substantial number of connections (1382 edges), suggesting a complex and highly interactive microbial network. Aeration tank has fewer nodes and edges, indicating a less complex network. Secondary sedimentation tank and sludge dewatering room display moderate complexity. In contrast, the fungal community (Figure 4B) across all sites shows a denser network with a higher number of nodes and edges, particularly at sludge dewatering room, which has the highest number of nodes (186) and edges (3235), suggesting a more intricate and potentially more resilient fungal community structure. These findings highlight the diversity and complexity of microbial interactions within bioaerosols, which can influence the stability and function of these communities in WWTPs. A pivotal finding was the central role of rare taxa in shaping the network architecture. In both bacterial and fungal networks, rare taxa constituted a substantial proportion of the nodes. More importantly, they were not peripheral entities but were extensively connected, forming numerous links both among themselves and with abundant taxa. The number of connections originating from or received by rare taxa often surpassed those within the abundant group alone, highlighting their integral position within the microbial community (Figure 4).

**Figure 5:**
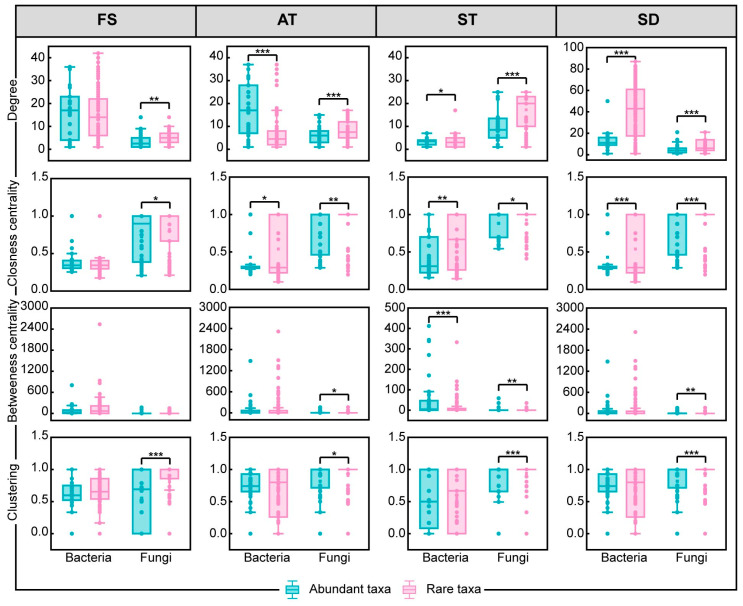
Comparison of node-level topological characteristics of abundant and rare taxa, including degrees, closeness centrality, betweenness centrality, and clustering. Significance levels are as follows: * *p* < 0.05, ** *p* < 0.01, and *** *p* < 0.001.

Network topology differed significantly between abundant and rare taxa (Figure 5). For both bacteria and fungi, abundant taxa consistently show higher degrees, indicating they are more connected within the network and likely play central roles in community interactions. Closeness centrality, which measures how close a node is to all other nodes, is also higher for abundant taxa, suggesting they are more influential in information flow and community dynamics. Betweenness centrality, which indicates how often a node lies on the shortest path between other nodes, is significantly higher for abundant taxa, highlighting their crucial role in connecting different parts of the network. Clustering coefficients, which measure the tendency of nodes to form clusters, are higher for abundant taxa, indicating they are more likely to be part of tightly knit groups [44,45]. These results suggest that abundant taxa are not only more prevalent but also structurally more important in shaping the network topology and functioning of microbial communities in WWTPs.

The findings from the co-occurrence network analysis and node-level topological characteristics provide valuable insights into the dynamics of microbial communities within bioaerosols at WWTPs. The distinct differences in network complexity and topological characteristics between abundant and rare taxa underscore the importance of considering the entire microbial spectrum when assessing community stability and function [40,46]. Abundant taxa, due to their higher connectivity and centrality, likely contribute significantly to the resilience and adaptability of microbial communities. Their presence may enhance the community’s ability to respond to environmental changes and maintain essential processes in WWTPs. However, the high connectivity of abundant taxa also suggests that they could rapidly spread or amplify under favorable conditions, potentially leading to operational challenges or increased health risks if they include pathogenic species. On the other hand, rare taxa, while less connected, may play niche roles that are crucial for maintaining biodiversity and ecosystem services. They could act as reservoirs of genetic diversity and contribute to community stability by filling specific ecological roles or providing ecosystem functions under certain conditions [18]. The variation in network characteristics across different sites highlights the influence of site-specific environmental factors and operational practices on microbial community structure and function [47].

It is important to note that co-occurrence patterns inferred from correlation-based network analysis do not necessarily equate to direct biological interactions (e.g., mutualism, competition). Correlations can arise from shared environmental preferences, dispersal limitations, or methodological artifacts [48,49]. Therefore, the links presented here represent potential ecological associations rather than confirmed mechanistic interactions. Notwithstanding these inherent limitations, co-occurrence network analysis remains a powerful tool for generating ecological insights. It helps identify taxa that occupy central topological positions (potential keystone taxa) and reveals the overall complexity and modularity of microbial communities [19]. The distinct network roles of abundant versus rare taxa highlighted in our study provide a testable ecological hypothesis regarding their differential contributions to community stability and function, which can be pursued through targeted mechanistic experiments in the future.

### 3.4. The Impact of Abundant and Rare Taxa on Pathogen Abundance

**Figure 6:**
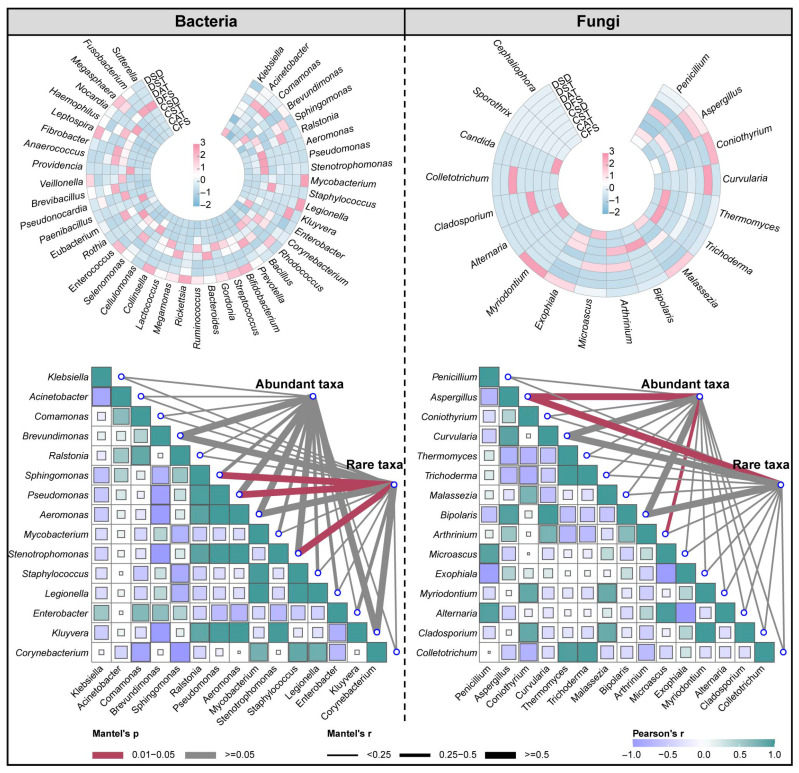
Pathogens composition at the genus level of aerosol bacterial and fungal community and the relation between the top 15 pathogens and abundant/rare taxa community structure.

A total of 45 and 18 pathogens were identified at the genus level of bacterial and fungal communities, respectively. In order to understand the driving factors for the differences in the abundance of these pathogens in different treatment units, the top 15 pathogens with relative abundance were selected for correlation analysis with the community structure of abundant and rare taxa (Figure 6). For fungal community, both abundant and rare taxa exhibited a significant correlation with the abundance of *Aspergillus* (*p* < 0.05). Significant correlations were only observed between rare bacterial taxa and multiple pathogen groups, including *Sphingomonas*, *Pseudomonas*, and *Stenotrophomonas* (*p* < 0.05), and there was no significant correlation between the community structure of abundant taxa and the abundance of pathogens. The significant associations between rare taxa and pathogens suggest rare taxa may serve as keystone species that disproportionately influence community structure and function through specific interactions with pathogens [19,50]. The absence of significant correlations between abundant bacterial taxa and pathogens suggests that the dominant bacterial community members either maintain neutral relationships with pathogens or that their influences are masked by the overwhelming signal of their abundance. This finding aligns with the concept that abundant taxa, while numerically dominant, may not necessarily be the primary drivers of specific ecological functions such as pathogen suppression or facilitation [37].

**Figure 7:**
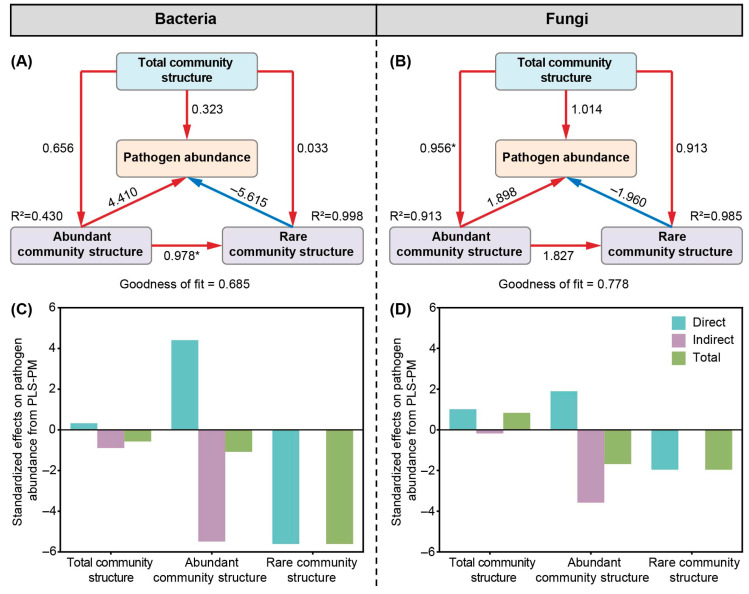
The direct and indirect effects of the abundant and rara taxa on pathogen abundance for aerosol bacterial (**A**,**C**) and fungal (**B**,**D**) community. Red and blue colors represent positive and negative effects, respectively. * *p* < 0.05

To disentangle the direct and indirect effects, PLS-PM was employed, unveiling a complex and contrasting influence of the two subcommunities on pathogen abundance (Figure 7). For the bacterial community, the path model demonstrated a strong positive direct effect of the abundant taxa on bacterial pathogen abundance (Path coefficient, 4.410; Figure 7A,C). In contrast, the rare taxa in the bacterial community exhibited a negative direct effect on pathogen abundance (Path coefficient, −5.615; Figure 7A,C). The model’s goodness of fit is 0.685, indicating a moderate level of explanation for the variance in pathogen abundance. As suggested by our network analysis, pathogens might be physically co-aggregated with abundant bacterial flocs or particles, leading to their coupled release and concentration in the air [51]. The significant negative direct effect of rare bacterial taxa on pathogen abundance, as revealed by the PLS-PM model, points to their potential antagonistic or suppressive role. Members of the rare biosphere may produce antagonistic compounds such as antibiotics, bacteriocins, or volatile organic compounds that directly inhibit the growth or viability of pathogens [12]. This aligns with the concept that the rare biosphere constitutes a reservoir of unique genetic and functional potential. Rare taxa could suppress pathogens through direct competition for essential nutrients, trace elements, or spatial niches within the bioaerosol particles or their source environments. This competitive exclusion is a fundamental driver of microbial community assembly [52]. The central topological positions of rare taxa in our co-occurrence networks support their potential role in stabilizing community structure. For the fungal community (Figure 7B,D), the total community structure has a strong positive direct effect on pathogen abundance (0.956), and the abundant community structure further amplifies this effect (1.898). The rare community structure also has a positive direct effect (1.827), but to a lesser extent. The model’s goodness of fit is 0.778, suggesting a high level of explanation for the variance in pathogen abundance. The significant positive effects of the abundant community structure on pathogen abundance in both bacterial and fungal communities suggest that interventions targeting the reduction in abundant taxa could be beneficial. However, the positive effects of the rare community structure in the fungal community indicate that a more nuanced approach is needed, as some rare taxa may contribute positively to pathogen control. This ecological perspective offers a novel and promising avenue for enhancing the environmental safety of wastewater treatment infrastructure.

## 4. Conclusions

In this study, the ecological characteristics of microbial abundant and rare groups in bioaerosols of sewage treatment plants were systematically analyzed, and their distinct roles in driving community structure and regulating pathogen dynamics were revealed. The main conclusions are as follows:

(1) Abundant taxa show a high degree of homogeneity in spatial distribution, and the community assembly is mainly dominated by stochastic processes. On the contrary, rare taxa exhibit high α diversity and significant heterogeneity shaped by deterministic, unit-specific environmental conditions.

(2) The co-occurrence network analysis showed that rare taxa were widely closely connected with abundant taxa and other rare taxa, occupying the key network topology position and being the potential keystone taxa.

(3) Abundant taxa exhibited a significant positive effect on pathogen abundance, whereas rare taxa demonstrated a significant negative effect for bacterial communities. This pattern indicates that pathogen risk in bioaerosols is not solely determined by the pathogens themselves, but is also profoundly influenced by the surrounding microbial community, particularly the complex interactions between taxa of different abundance levels.

In summary, the findings emphasize that future assessments of the ecological and health risks posed by these bioaerosols must concurrently consider the carrier effect of abundant taxa and the regulatory potential of rare taxa. The specific composition or ratio of abundant taxa (particularly bacteria) can serve as a sentinel indicator. Their abnormal proliferation may signal a higher risk of pathogen transmission, necessitating the initiation of enhanced surveillance or intervention measures. At the level of risk control, physical or chemical methods could be explored to selectively suppress the generation and dispersal of dominant microbial groups in aerosols, and it is crucial to identify key rare taxa with strong antagonistic potential and investigate how to modulate environmental factors to selectively enrich these beneficial rare taxa.

While translating these ecological insights into standard engineering practices requires further validation, our work provides a critical scientific foundation and a clear roadmap for developing next-generation, ecologically informed strategies for proactive bioaerosol risk management in WWTPs. Furthermore, the sampling campaign was conducted in summer, and thus the potential influences of seasonal variations on the dynamics of abundant and rare taxa in bioaerosols were not captured. Future long-term monitoring across different seasons and under varied weather conditions is warranted to validate the temporal stability and generalizability of the patterns observed here.

## Data Availability

The original contributions presented in the study are included in the article, further inquiries can be directed to the corresponding author.
